# The prognostic and potentially immunomodulatory role of cartilage oligomeric matrix protein in patients with gastric and esophageal adenocarcinoma

**DOI:** 10.1007/s00262-024-03656-y

**Published:** 2024-04-02

**Authors:** Konstantinos S. Papadakos, Gilar Gorji-Bahri, Chrysostomi Gialeli, Charlotta Hedner, Catharina Hagerling, Maria C. Svensson, Martin Jeremiasen, David Borg, Richard Fristedt, Karin Jirström, Anna M. Blom

**Affiliations:** 1 Division of Medical Protein Chemistry, Department of Translational Medicine, Lund University, Inga Maria Nilsson’s Street 53, 214 28 Malmö, Sweden; 2 Cardiovascular Research - Translational Studies, Department of Clinical Sciences, Lund University, Malmö, Sweden; 3 Department of Clinical Sciences Lund, Oncology and Therapeutic Pathology, Lund University, Lund, Sweden; 4 Department of Experimental Medical Science, Lund University, Lund, Sweden; 5 Department of Clinical Sciences Lund, Surgery, Lund University, Lund, Sweden

**Keywords:** COMP, Gastric and esophageal adenocarcinoma, Collagen, Fibrosis, Immune cells

## Abstract

**Background:**

Cartilage oligomeric matrix protein (COMP) is a novel regulator of the tumor microenvironment. Studies in colon cancer and pancreatobiliary adenocarcinoma have revealed COMP expression to be associated with decreased infiltration of immune cells in the tumor microenvironment. Herein, the expression of COMP was investigated in gastric and esophageal adenocarcinoma with particular reference to its the relationship with the immune microenvironment.

**Methods:**

COMP expression was evaluated in tissue microarrays representing primary tumors from 159 patients with chemo- and radiotherapy naïve esophageal and gastric adenocarcinoma and 67 matched samples of lymph node metastases using immunohistochemistry. Additionally, collagen fibers were stained with Sirius Red and evaluated with the FIJI macro TWOMBLI algorithm.

**Results:**

The expression of COMP in cancer cells in the entire cohort was associated with shorter overall survival (OS) (*p* = 0.013) and recurrence-free survival (RFS) (*p* = 0.029), while COMP expression in the stroma was correlated with shorter RFS (*p* = 0.042). Similar correlations were found for patients with gastric adenocarcinoma, whereas COMP expression was not prognostic in esophageal adenocarcinoma. Further, in the entire cohort, the expression of COMP in the stroma was correlated with exclusion of different populations of immune cells (CD8^+^, CD3^+^, FoxP3^+^, CD20^+^) from the tumor microenvironment. Finally, higher density and alignment of collagen fibers were correlated with the expression of COMP in the stroma.

**Conclusions:**

Expression of COMP in gastric and esophageal adenocarcinoma was correlated with shorter OS and RFS. A reduced number of immune cells infiltrated the tumor microenvironment when COMP expression was detected. This phenomenon could be attributed to the denser collagen deposits, a hallmark of tumor fibrosis observed in COMP-expressing tumors.

**Supplementary Information:**

The online version contains supplementary material available at 10.1007/s00262-024-03656-y.

## Introduction

Understanding the mechanisms of resistance to immunotherapy in different types of cancers will allow for the usage of novel effective treatments by providing new targets that can enhance the effect of immunotherapy.

Gastric and esophageal adenocarcinoma share similar etiopathological characteristics. Both types of cancer may arise due to chronic inflammation, which leads to epithelial transformation of the normal mucosa. In esophageal adenocarcinoma, Barrett’s esophagus develops as a result of gastroesophageal reflux disease, which can lead to dysplasia and, finally, adenocarcinoma [1]. Similarly, in gastric cancer, chronic inflammation, mainly due to *Helicobacter pylori* infection, leads to chronic gastritis, atrophic gastritis followed by dysplasia, and transformation into gastric adenocarcinoma [2, 3].

Cartilage oligomeric matrix protein (COMP) is a newly reported regulator of the tumor microenvironment. It is primarily expressed in the cartilage of healthy individuals, playing a crucial role in normal extracellular matrix organization, vital for cartilage stiffness and integrity [4]. COMP has been shown to be upregulated in breast cancer, both in tumor cells and in the stroma, and high expression in tumor cells was an independent factor of shorter survival [5]. In line with these results, the serum level of COMP has also been shown to be an independent predictive factor of breast cancer patients’ survival comparable with classical prognostic markers, such as estrogen receptor (ER), progesterone receptor (PR) and human epidermal growth factor receptor (HER2) [6]. The underlying molecular mechanism involves the binding of COMP to the Notch3 receptor and its ligand Jagged1, thereby bridging them. The formation of this protein complex leads to the activation of the Notch pathway and, consequently, a higher proportion of the cancer stem cell population in the tumor [7]. Furthermore, COMP expression by cancer cells affects the cancer cell metabolism, increased resistance to apoptosis, and enhanced tumor metastasizing ability [5]. Similar associations of COMP expression being related to survival and time to recurrence have been found in studies on patients with colon cancer [8], hepatocellular carcinoma [9], and urothelial carcinoma [10].

A recent study revealed that COMP is highly expressed in pancreatobiliary type periampullary adenocarcinoma, which was associated with the exclusion of CD8^+^ T-cells from the cancer cell compartment [11]. High levels of COMP were also correlated with denser collagen fibers in the tumor stroma, indicating a stiffer and fibrotic tumor microenvironment. The exclusion of immune cells from the cancer cell compartment by a stroma dense in collagen fibers is a known mechanism of resistance to PD-L1 inhibitor therapy [12]. Accordingly, a newly published study revealed a similar phenomenon of immune cells exclusion from the tumor in patients with colorectal cancer [13]. Tumors expressing high levels of COMP had a decreased number of infiltrating immune cells in the tumor microenvironment. Furthermore, high levels of COMP expression were associated with lower levels of PD-L1 expression by both cancer cells and immune cells.

In this study, we aimed to investigate the association of COMP expression with infiltrating immune cells and the organization of collagen fibers in esophageal and gastric adenocarcinoma. Furthermore, we examined the associations of COMP expression in cancer cells and in the stroma, respectively, with overall survival (OS) and recurrence-free survival (RFS). Additionally, COMP expression was evaluated in relation to clinicopathological characteristics, and compared between primary tumors and local lymph node metastases.

## Methods

### Cohort

Patients included in the current study are part of a cohort that has been characterized previously [14–17]. In total, 174 patients with chemotherapy and radiotherapy naïve esophageal and gastric adenocarcinoma, subjected to surgical resection between 31 December 2010 and 1 January 2016 at the Lund and Malmö University Hospitals were included. Clinicopathological data were obtained retrospectively from medical records. The follow-up started from the day of surgery up until March 2016. The Ethics Committee of Lund University (No. 445/07) approved the study, whereby the committee waived no need for consent other than the option to opt out. All patient data were anonymized and de-identified prior to analysis.

### Immunohistochemistry staining

Tumor tissue microarrays (TMA) were assembled as described [14, 18], sliced and antigens retrieved with citric acid buffer at pH 6. For the majority of the patients, two tumor samples were included. For 80 patients, a sample from lymph node metastasis was also stained. Slides were stained overnight with rabbit polyclonal anti-COMP antibody characterized for its specificity previously [5]. Stained slides were scanned using an Aperio Scanner (Leica) at 40X. COMP expression by the cancer cells and in the stroma was evaluated by four researchers blindly scoring from 0 (negative expression) to 3 (highest expression). The total percentage of COMP positive cells present in both cancer cells and the stroma was also evaluated using the Qupath software [19]. The infiltrating immune cells, PD-L1 and PD-1 expression were evaluated in previous studies [14–17]. Samples exhibiting absence of nuclear staining for MLH1, PMS2, MSH2, or MSH6 were identified as having deficient mismatch repair (dMMR) status [16].

### Collagen staining

Slides of TMAs were stained for collagen with 0.1% Sirius Red (Sigma-Aldrich) and non-collagenous proteins with 0.04% Fast Green (Merck) staining solution in saturated picric acid. Cells nuclei were stained with Weigert's hematoxylin nuclear staining solution (Histolab). The intensity of collagen was assessed utilizing the QuPath open software, and collagen organization was evaluated with FIJI macro TWOMBLI [20].

### Statistics

When two parameters were compared, the p-values were calculated with χ^2^ two-tailed test. Accordingly, when three parameters were compared, Kruskal–Wallis test was used. Survival was assessed with Kaplan–Meier analysis and log rank test. We used the Cox proportional hazards model to investigate the association between predictor variables and patient survival. Pairs of matched samples from the primary tumors and lymph node metastases were evaluated for dependency with McNemar's test. The calculations were performed with IBM SPSS Statistics for Macintosh, Version 29.0.0 and graphs were prepared with Prism 10.

## Results

### The presence of COMP in the stroma correlates with local lymph node metastases

**Fig. 1:**
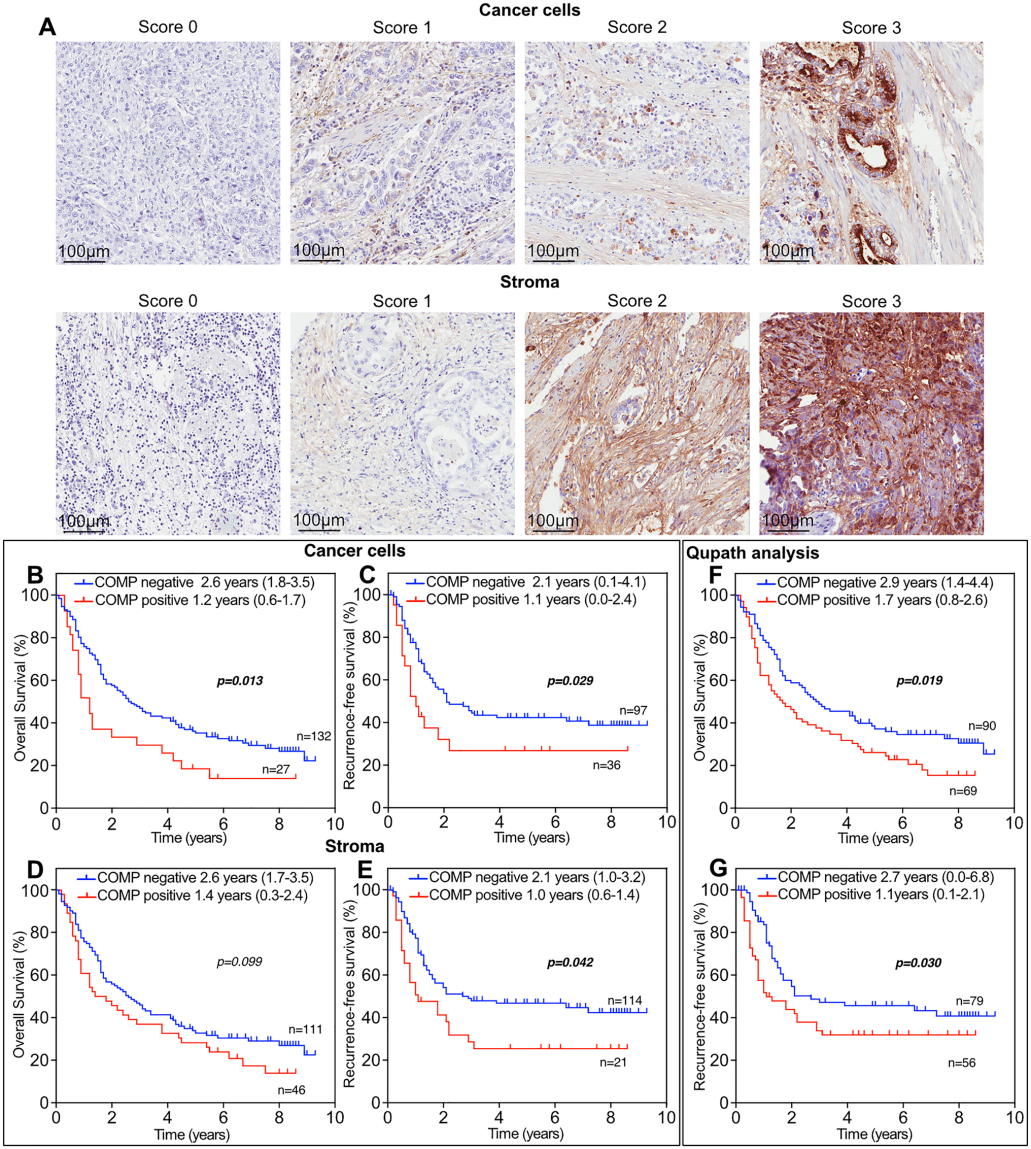
COMP expression in patients with esophageal and gastric adenocarcinoma. **A** Representative images of primary tumors stained for COMP expression and evaluated by blinded observers. Different levels of COMP expression were detected in cancer cells and the stroma. Patients’ overall survival (**B** and **D**) and recurrence-free survival **(C** and **E**) were estimated for the entire cohort using Kaplan–Meier analysis. COMP −: patients with a score of 0; COMP+: patients with a score of 1–3. **F** and **G** COMP expression in tissue biopsies (including cancer cells and stroma) was also evaluated with Qupath software. The total percentage of COMP-positive cells in tumor and stroma was calculated. COMP −: patients with tumors in which less than 1% of cells expressed COMP; COMP+: patients with tumors in which more than 1% of cells expressed COMP. Significant *p* < 0.05 values are depicted in bold. Values in parenthesis represent the 95% confidence interval

COMP expression was evaluated in 159 of 174 samples, as the omitted TMA cores had been detached or lacked cancer cells or stroma. The samples were divided into two groups: COMP negative (score 0) and COMP positive (score1-3; Fig. 1A). In the entire cohort, the presence of COMP in the stroma was associated with the location of the primary tumor (*p* < 0.001) and a more advanced N-stage (*p* = 0.005). In patients with esophageal adenocarcinoma, stromal COMP expression was not correlated with any clinicopathological characteristic (Table S1). In patients with gastric adenocarcinoma, COMP expression in both cancer cells and the stroma was associated with R-status (cancer cells *p* = 0.002; stroma *p* = 0.015), and COMP expression in cancer cells was associated with T-stage (*p* = 0.046) (Table S2).

### COMP expression is decreased in lymph node metastases

McNemar test was applied to compare COMP expression in tumor cells in pairs of primary tumors and lymph node metastases (n = 67). This showed that 2 pairs had gained COMP expression, 4 pairs had retained expression, and 13 pairs had lost the expression of COMP in lymph node metastases (*p* = 0.007; Table S3). Comparison of the expression of COMP in the stroma of the primary tumors with the expression of cancer cells in the lymph node metastasis showed that 2 pairs had gained expression, 4 pairs had retained expression, and 26 pairs had lost the expression of COMP in lymph node metastases (*p* < 0.001; Table S3).

### Expression of COMP correlates with reduced survival and time to recurrence

COMP expression was scored manually in the cancer cells and in stroma, respectively. Patients with tumors expressing COMP in cancer cells had a median OS of 1.2 years compared with 2.6 years for patients with tumors lacking COMP expression in cancer cells (*p* = 0.013; Fig. 1B), and the corresponding figures for RFS were 1.1 years and 2.1 years, respectively (*p* = 0.029; Fig. 1C). COMP expression in the tumor stroma did not correlate with OS (*p* = 0.099; Fig. 1D) but with a shorter RFS (*p* = 0.042; Fig. 1E). In addition, total COMP expression in tumors (including cancer cells and stroma) expressed as percentage of positive cells was analysed using Qupath software. The samples were dichotomized based on COMP expression (< 1% and ≥ 1% of COMP positive cells). This analysis confirmed the association of COMP expression with OS and RFS.

**Fig. 2:**
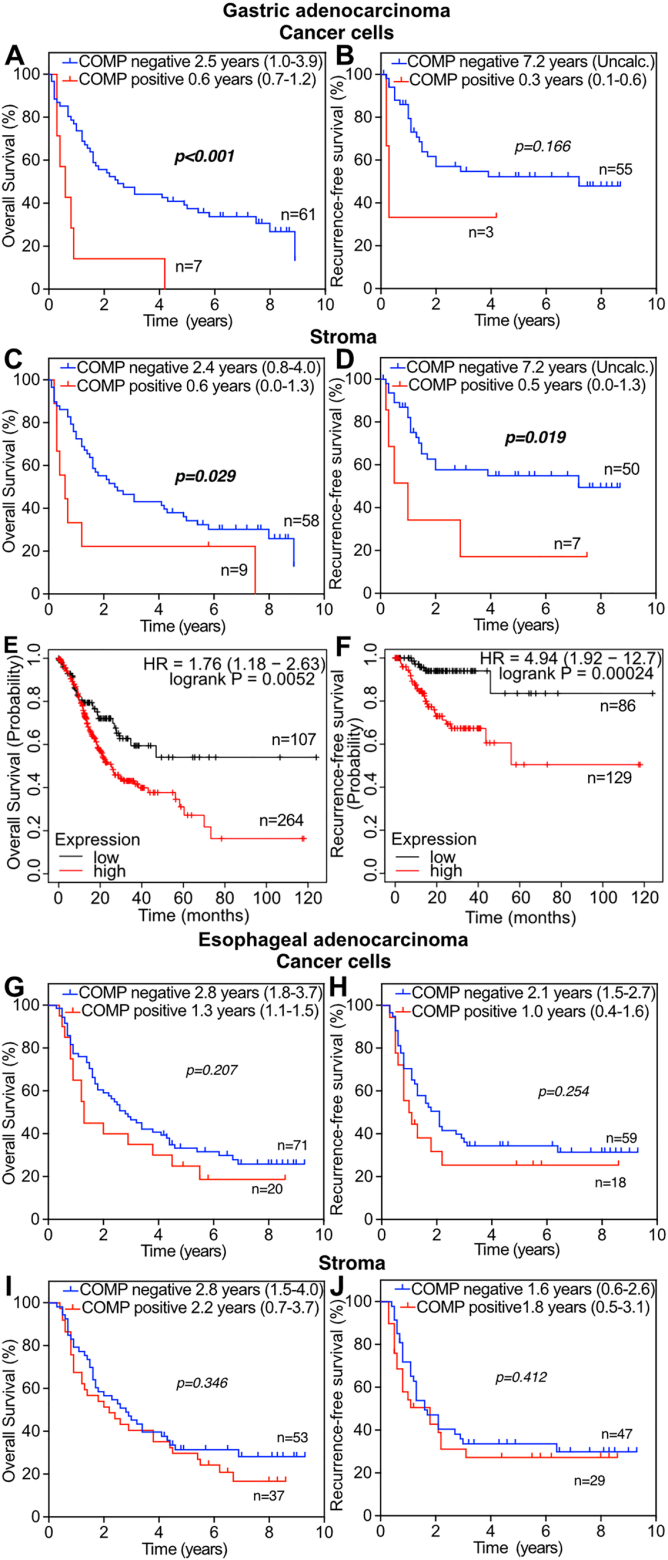
Overall survival and recurrence-free survival were calculated **(A–D)** in relation to scored COMP expression in patients with gastric adenocarcinoma. mRNA expression levels of COMP in tumors from patients with gastric adenocarcinoma were analyzed using the Kaplan–Meier plotter online tool, correlated with overall survival **(E)** and recurrence-free survival **(F)**. Overall survival and recurrence-free survival for patients with esophageal adenocarcinoma **(G–J)** according to Kaplan–Meier analysis. COMP−: patients with a score of 0; COMP+: patients with a score of 1–3; Uncal.: incalculable. Significant *p* < 0.05 values are depicted in bold. Values in parenthesis represents the 95% confidence interval

When patients were stratified by primary tumor location, gastric adenocarcinoma patients with tumors with COMP expression in cancer cells had a median OS of 0.6 years, compared with 2.5 years for patients with tumors without COMP expression in cancer cells (*p* < 0.001; Fig. 2A and B). Similarly, patients with COMP expression in the tumor stroma had a median OS of 0.6-year (*p* = 0.029; Fig. 2C) and a median RFS of 0.5 years, (*p* = 0.019; Fig. 2D) compared to 2.4 and 7.2 years respectively, in patients with COMP-negative tumor stroma. To further evaluate this observation, RNA-sequencing data of patients with gastric adenocarcinoma (OS n = 371, RFS n = 215) were evaluated using the online tool, Kaplan Meier plotter [21]. Patients with high levels of COMP expression in tumors had shorter median OS (*p* = 0.005; Fig. 2E) and RFS (*p* < 0.001; Fig. 2F) compared with patients expressing low levels of COMP. In contrast, the presence of COMP in cancer cells or stroma was not prognostic in patients with esophageal adenocarcinoma (Fig. 2G–J) (Table 1).

**Table 1:** Associations of COMP expression with clinicopathological characteristics in the entire cohort

Factor	Cancer cells					Stroma				
	COMP negative		COMP positive		*p* value	COMP negative		COMP positive		*p* value
All (N = 159)	N	(%)	N	(%)		N	(%)	N	(%)	
Age at diagnosis					0.125					0.570
< 50	6	3.8%	0	0.0%		5	3.2%	1	0.6%	
50–70	57	35.8%	17	10.7%		49	31.2%	24	15.3%	
> 70	69	43.4%	10	6.3%		57	36.3%	21	13.4%	
Sex					0.654					0.288
Female	29	18.2%	7	4.4%		28	17.8%	8	5.1%	
Male	103	64.8%	20	12.6%		83	52.9%	38	24.2%	
Location					0.052					** < 0.001**
Esophagus	71	44.7%	20	12.6%		53	33.8%	37	23.6%	
Stomach	61	38.4%	7	4.4%		58	36.9%	9	5.7%	
Adjuvant therapy					0.793					0.442
No adjuvant	124	78.0%	25	15.7%		105	66.9%	42	26.8%	
Adjuvant	8	5.0%	2	1.3%		6	3.8%	4	2.5%	
T-stage					0.107					0.097
pT1–T2	39	24.8%	4	2.5%		33	21.3%	8	5.2%	
pT3–T4	91	58.0%	23	14.6%		76	49.0%	38	24.5%	
N-stage					0.112					**0.005**
pN0	45	28.3%	5	3.1%		42	26.8%	7	4.5%	
pN1–3	87	54.7%	22	13.8%		69	43.9%	39	24.8%	
M-stage					0.307					0.549
M0 or Mx	117	73.6%	22	13.8%		98	62.4%	39	24.8%	
M1	15	9.4%	5	3.1%		13	8.3%	7	4.5%	
Grade					0.510					0.948
Low	87	54.7%	16	10.1%		73	46.5%	30	19.1%	
High	45	28.3%	11	6.9%		38	24.2%	16	10.2%	
R-status					0.151					0.055
R0	94	59.1%	15	9.4%		82	52.2%	25	15.9%	
R1	33	20.8%	9	5.7%		24	15.3%	18	11.5%	
R2	5	3.1%	3	1.9%		5	3.2%	3	1.9%	
Laurén					0.345					0.569
Intestinal	89	56.0%	22	13.8%		76	48.4%	34	21.7%	
Mixed	7	4.4%	1	0.6%		5	3.2%	3	1.9%	
Diffuse	36	22.6%	4	2.5%		30	19.1%	9	5.7%	
Vascular invasion					0.356					0.295
V0	15	22.4%	0	0.0%		14	21.2%	1	1.5%	
V1	45	67.2%	6	9.0%		38	57.6%	12	18.2%	
Uncertain	1	1.5%	0	0.0%		1	1.5%	0	0.0%	
MMR					0.347					0.916
pMMR	119	75.3%	26	16.5%		101	64.7%	42	26.9%	
dMMR	12	7.6%	1	0.6%		9	5.8%	4	2.6%	

*Abbeviations* COMP, cartilage oligomeric matrix protein MMR, DNA mismatch repair; pMMR, proficient MMR; dMMR, deficient MMR. The bold indicates *p* values < 0.05, Calculated with χ^2^ two-tailed exact *p *value

### The prognostic value of COMP is independent of conventional prognostic markers

We subsequently examined the prognostic significance of COMP expression in multivariable Cox regression analysis. In the entire cohort (n = 159), COMP expression by cancer cells was a negative prognostic marker for OS (*p* = 0.036) independently of primary tumor location, TNM-stage, tumor grade and Laurén type (Table 2). Furthermore, in the entire cohort, COMP expression by cancer cells served as a prognostic marker of shorter OS (*p* = 0.015) and RFS (*p* = 0.027), independently of PD-L1 expression on cancer cells and immune cells, PD-1 expression on immune cells, and MMR status (Table 3).

**Table 2:** Cox multivariable analyses adjusted for conventional prognostic factors

Variable	Cancer cells				Stroma			
	HR	95% CI		*p* value	HR	95% CI		*p* value
Survival								
COMP (0 vs. 1–3)	1.687	1.036	2.747	**0.036**	1.246	.803	1.934	0.326
Location (Esophagus vs. Stomach)	1.454	0.946	2.235	0.088	1.452	.926	2.276	0.104
T-stage (pT1-T2 vs. pT3-T4)	1.908	1.188	3.065	**0.008**	1.853	1.152	2.978	**0.011**
N-stage (pN0 vs. pN1-3)	2.017	1.258	3.233	**0.004**	2.077	1.291	3.340	**0.003**
M-stage (M0 or Mx vs. M1)	2.023	1.218	3.361	**0.007**	1.930	1.162	3.205	**0.011**
Grade (Low vs. high)	1.376	0.891	2.124	0.150	1.306	.840	2.030	0.235
Laurén (Intestinal vs. mixed or diffuse)	1.232	0.794	1.912	0.351	1.264	.813	1.967	0.298
Recurrence-free survival								
COMP (0 vs. 1–3)	1.346	0.720	2.518	0.352	1.392	0.814	2.381	0.227
Location (Esophagus vs. Stomach)	0.987	0.571	1.703	0.961	1.016	0.591	1.746	0.954
T-stage (pT1-T2 vs. pT3-T4)	3.162	1.579	6.330	**0.001**	3.119	1.548	6.283	**0.001**
N-stage (pN0 vs. pN1-3)	7.780	3.269	18.515	** < 0.001**	7.945	3.342	18.889	** < 0.001**
M-stage (M0 or Mx vs. M1)	1.508	0.780	2.917	0.222	1.382	0.708	2.698	0.343
Grade (Low vs. High)	2.011	1.114	3.629	**0.020**	1.944	1.084	3.485	**0.026**
Laurén (Intestinal vs. mixed or diffuse)	1.668	0.971	2.865	0.064	1.764	1.022	3.044	**0.042**

*Abbeviations* COMP, cartilage oligomeric matrix protein. The bold indicates *p *values < 0.05

**Table 3:** Cox multivariable analyses of the entire cohort adjusted for immune checkpoint related proteins

Variable	Cancer cells				Stroma			
	HR	95% CI		*p *value	HR	95% CI		*p* value
Survival								
COMP (0 vs. 1–3)	1.779	1.120	2.825	**0.015**	1.265	0.843	1.899	0.256
PD-L1 cancer cells	1.533	0.997	2.359	0.052	1.547	1.005	2.381	**0.047**
PD-L1 immune cells	0.683	0.486	0.960	**0.028**	0.709	0.502	1.002	0.051
PD-1 immune cells	0.672	0.463	0.978	**0.038**	0.703	0.478	1.034	0.074
pMMR vs. dMMR	1.315	0.688	2.511	0.408	1.176	0.611	2.264	0.628
Recurrence-free survival								
COMP (0 vs. 1–3)	1.906	1.075	3.380	**0.027**	1.607	0.983	2.629	0.058
PD-L1 cancer cells	1.777	1.064	2.970	**0.028**	1.709	1.022	2.858	**0.041**
PD-L1 immune cells	0.680	0.433	1.068	0.094	0.727	0.460	1.147	0.170
PD-1 immune cells	0.543	0.334	0.881	**0.013**	0.600	0.369	0.977	**0.040**
pMMR vs. dMMR	0.513	0.177	1.490	0.220	0.440	0.150	1.290	0.135

*Abbeviations* COMP, cartilage oligomeric matrix protein; PD-L1, programmed death-ligand 1; PD-1, Programmed cell death protein; MMR, DNA mismatch repair; pMMR, proficient MM; dMMR, deficient MMR. The bold indicates *p* values < 0.05

### The expression of COMP is associated with a reduced number of immune cells in the tumor microenvironment

**Fig. 3:**
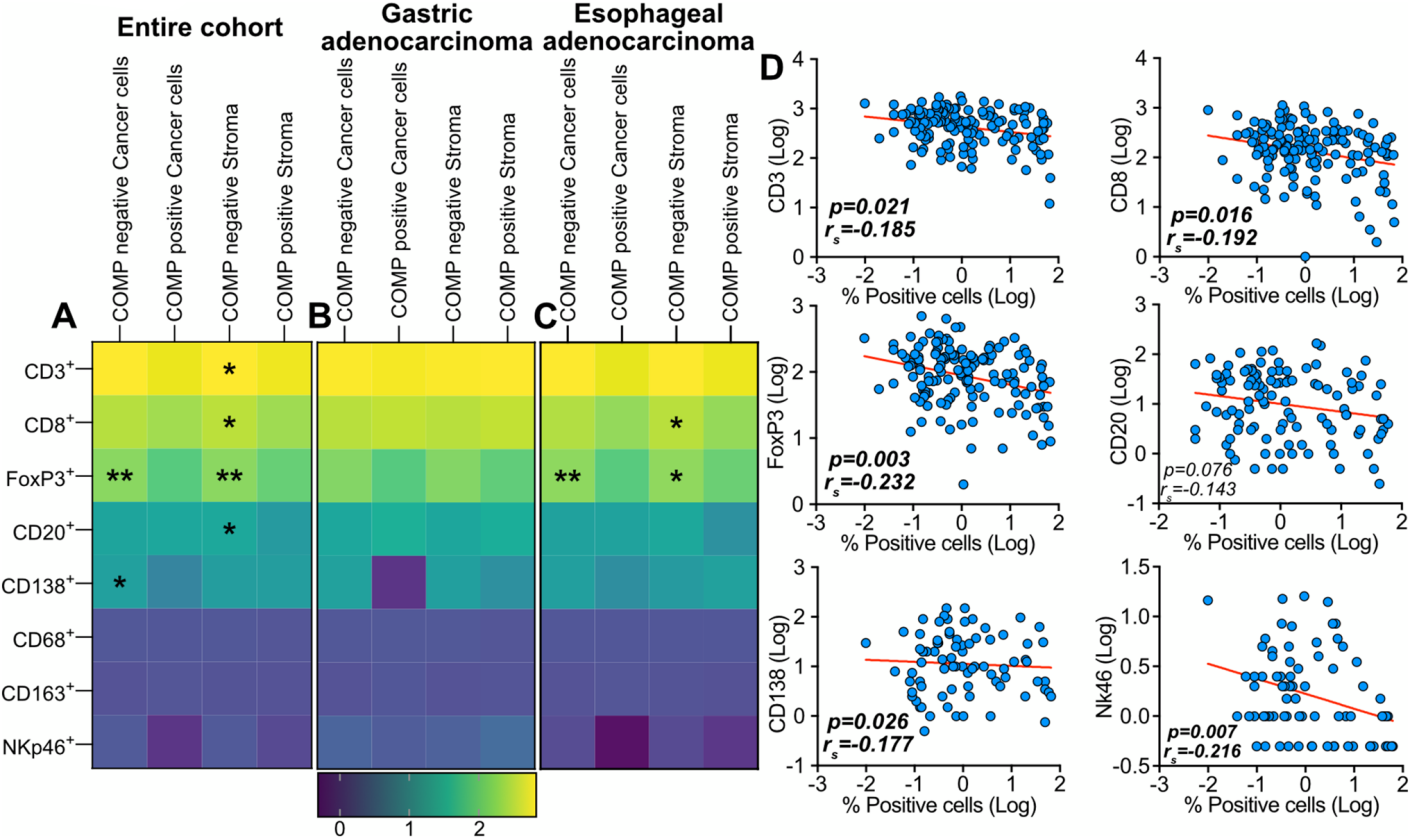
The presence of specific immune cell populations in the tumor samples was correlated with the expression of COMP by cancer cells and in the stroma in the entire cohort **(A)** and the subgroups of gastric **(B)** and esophageal adenocarcinoma **(C)**. COMP-: patients with a score of 0; COMP+ : patients with a score of 1–3. **(D)** Similar results were observed when the quantity of infiltrating immune cells correlated with the total percentage (including cancer cells and stroma) of COMP-positive cells, analyzed by the QuPath software on TMAs. COMP−: patients with tumors in which less than 1% of cells expressed COMP; COMP+ : patients with tumors in which more than 1% of cells expressed COMP. Spearman’s regression analysis used for the evaluation of the statistical correlations; *p ≤ 0.05, **p ≤ 0.01, ***p ≤ 0.001 and ****p ≤ 0.0001

The presence of infiltrating immune cells in tumor samples has been previously well characterized for the current cohort [14, 15]. Here, we found that in the whole cohort, COMP expression in the stroma was associated with fewer infiltrating CD3^+^ T-cells (*p* = 0.043), CD8^+^ T-cells (*p* = 0.034), activated FoxP3^+^ T-cells (*p* = 0.008), and B-cells (CD20^+^, *p* = 0.047; Fig. 3A). COMP expression in the cancer cells was also associated with less infiltrating CD138^+^ B-cells (*p* = 0.038) and activated FoxP3^+^ T-cells (*p* < 0.001). On the other hand, in patients with gastric adenocarcinoma, no statistically significant differences were observed regarding infiltrating immune cells (Fig. 3B). In contrast, patients with esophageal adenocarcinoma had fewer infiltrating CD8^+^ T-cells (*p* = 0.042) when COMP was expressed in the stroma (Fig. 3C). Similarly, less activated FoxP3^+^ T-cells (cancer cells: *p* = 0.001, stroma: *p* = 0.020) infiltrated the tumors when COMP was expressed by both cancer cells and stroma. Furthermore, similar observations regarding infiltrating immune cells were made across the entire cohort when COMP expression in tumors (including cancer cells and stroma) was evaluated using the Qupath software (Fig. 3D). Expression of COMP was not correlated with the expression of PD-L1 by the cancer cells or immune cells (Table S4). Furthermore, PD-1 expression by the immune cells did not correlate with the expression of COMP.

### Presence of COMP in the stroma is associated with denser collagen fibers.

**Fig. 4:**
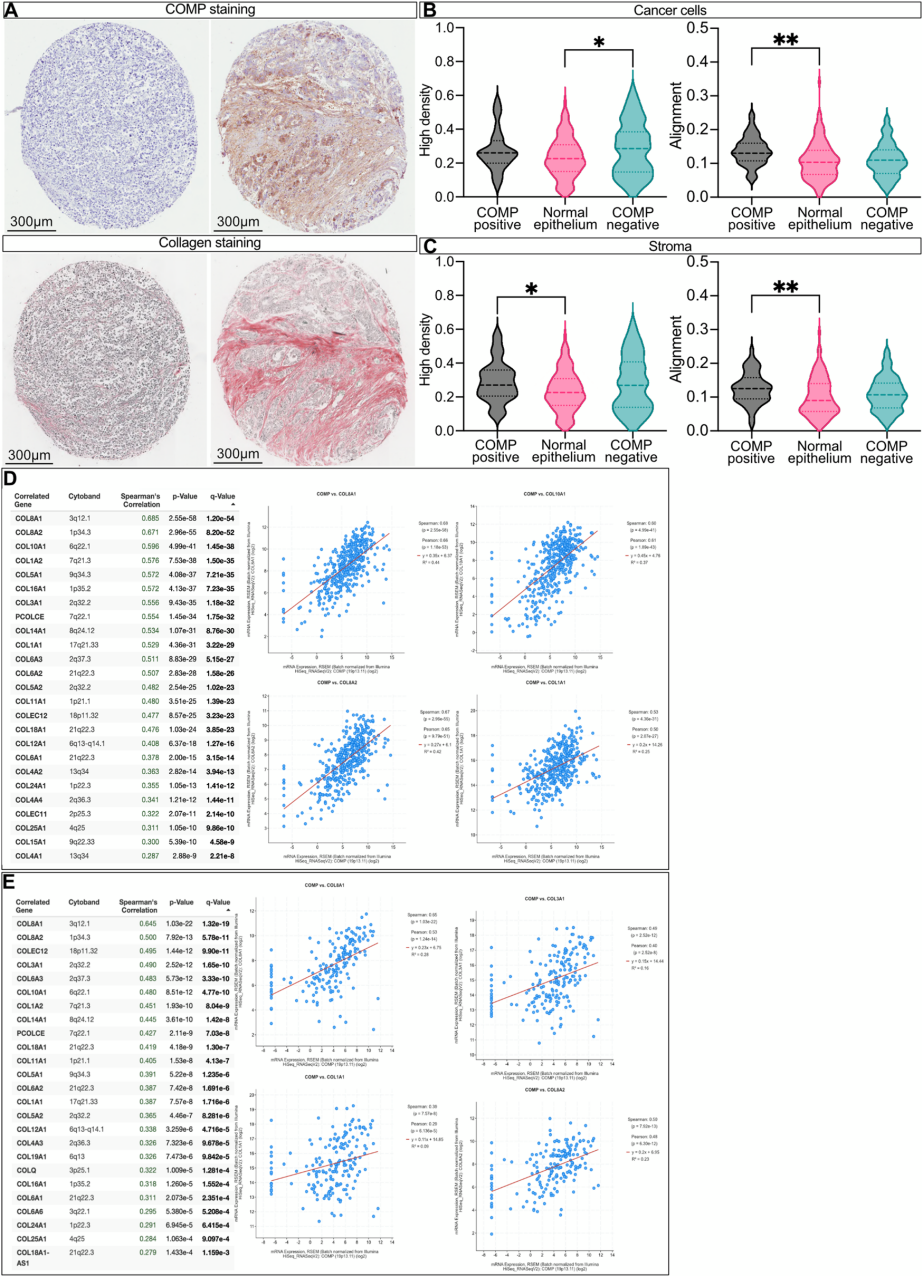
TMAs were stained with the Sirius red method for collagen expression (**A**). The organization and the density of the collagen fibers were evaluated with FIJI macro TWOMBLI, correlated with the expression of COMP by the cancer cells (**B**) and in stroma (**C**). mRNA sequencing data of patients with gastric (**D**) or esophageal (**E**) adenocarcinoma were retrieved from TCGA, PanCancer Atlas cohort using cBioPortal, showing the correlation of COMP expression with several collagen genes’ expression. Kruskal–Wallis non-parametric test was used for the evaluation of the statistical correlations; *p ≤ 0.05, **p ≤ 0.01, ***p ≤ 0.001 and ****p ≤ 0.0001

The collagen fibers’ density and organization were evaluated and compared with COMP expression (Fig. 4A). The presence of COMP in the stroma was correlated with higher density and alignment of collagen fibers compared with the normal tissue levels of collagen (Fig. 4B&C). To further confirm this observation, the mRNA sequencing data of patients with gastric (Fig. 4D) or esophageal adenocarcinoma (Fig. 4E) were retrieved from TCGA, PanCancer Atlas cohort [22] using cBioPortal. Data analysis revealed that the expression of COMP in the tumors was correlated with the expression of several types of collagens. Moreover, gene pathway analysis was performed on the same patient samples using cBioPortal tools. Pathways previously identified as being involved in the oncogenic effect of COMP in other types of cancer, such as Notch, TGF-β, WNT, PI3K were found to be affected. Additionally, new pathways have been identified, including Hippo and RTK-RAS (Fig. S1).

## Discussion

The current study demonstrated that COMP is expressed in the tumors of patients with gastric or esophageal adenocarcinoma. Expression of COMP was associated with poorer OS and RFS of patients in the entire cohort. Fewer immune cells were infiltrating the tumors when COMP expression was detected mainly in stroma. In addition, denser collagen fibers were present in the tumor microenvironment when COMP was detected in the tumor stroma.

The presence of COMP in the stroma was associated with different clinicopathological characteristics of the patients and RFS in the entire cohort. These results are interesting as the initial published studies indicated that the levels of COMP expression by the cancer cells rather than in stroma were predictive of disease outcome in breast cancer and prostate cancer [5, 23]. We recently reported that high levels of COMP expression both by the cancer cells and in the stroma was a predictive factor for OS and RFS in patients with intestinal type periampullary adenocarcinoma [11] and colorectal cancer [13]. Collectively, these observations indicate that the topology of COMP expression and its prognostic value is cancer-specific and might depend on the pathobiology of the tumor type. For instance, in ovarian cancer, the contribution of stroma in tumor progression is pivotal. Interestingly, COMP was the most upregulated protein when the stroma protein content of high-grade serous ovarian carcinoma was characterized with proteomic analysis [24]. Notably, COMP was prognostic in patients with gastric adenocarcinoma, but in contrast, it was not prognostic in patients with esophageal adenocarcinoma. These results might be attributed to the small sample size. Future studies need to be conducted to clarify the prognostic role of COMP in patients with gastric adenocarcinoma.

The analysis of COMP expression in pairs of primary tumors and lymph nodes with metastasis showed that most primary tumors lost COMP expression when the cancer cells metastasized to local lymph nodes. These results, combined with the observation that tumors expressing COMP formed more distant metastases [5, 6], led us to hypothesize that COMP expression in the primary tumor contributes to the initial stages of metastasis even by promoting local tissue invasion or by facilitate the intravasation. Indeed, one study reported that COMP promotes the induction of epithelial-mesenchymal transition in colorectal cancer [25]. Future studies need to clarify if this is only a mechanism that observed in cancer of the gastrointestinal tract, or if it is universally observed in COMP expressing tumors.

We observed that the expression of COMP by cancer cells was a prognostic marker of patients' OS and RFS. Intriguingly, this observation also holds true when adjusted for markers of immune checkpoint inhibitor response, such as PD-L1 and MMR status. This may suggest a potentially independent mechanism for the exclusion of immune cells from the tumor microenvironment. One known mechanism of resistance to immune checkpoint inhibitors therapy is the exclusion of T-cells, which was initially observed in a study investigating patients with urothelial cancer who participated in a clinical trial of anti-PD-L1 therapy. Here, the accumulation of collagen in the tumor stroma, found in patients resistant to the therapy, led to the exclusion of CD8^+^ T-cells access to the cancer cells [12]. COMP is mainly expressed in cartilage, participating in the organization of ECM structure by binding to collagen fibers and consequently contributing to cartilage stiffness [4]. Also, COMP aids the expression and secretion of collagen, acting as a secretory chaperon [26]. Expression of COMP is also evident in fibrotic tissues such as skin in scleroderma, where the expression of the protein intensifies fibrosis and renders tissue stiffer [27–29]. Interestingly, extracellular matrix stiffness has been associated with cancer cell invasion and metastasis in several types of cancers [30–32]. In particular, the higher matrix stiffness was correlated with more aggressive types of breast cancer [33, 34]. In the current study, we observed less infiltrating T-cells and other immune cells in the COMP-expressing tumors. Moreover, the COMP-expressing tumors had a higher density of collagen fibers. Thus, COMP expression may contribute to immune checkpoint inhibitors resistance by immune exclusion of T-cells from the tumor compartment. This observation is in line with our recent studies showing a correlation between COMP expression and less infiltrating immune cells in the tumors and higher levels of tumor fibrosis in pancreatobiliary type periampullary adenocarcinoma [11] and colorectal cancer [13]. Interestingly, the correlation of COMP, and in general the wider family of thrombospondin proteins, with reduced infiltration of immune cells in gastric tumors has been predicted by a bioinformatic study [35]. Additionally, it was observed that COMP expression associated with a higher alignment of collagen fibers in breast cancer tumors. Collagen fiber alignment was found to promote the exclusion of T-cells from infiltrating the tumor microenvironment [36]. Nonetheless, a limitation of the study is that we cannot exclude that COMP can directly bind to the immune cells and modulate their activity. Indeed, it was reported that COMP binds to *M. catarrhalis* and can prevent the phagocytosis and killing by neutrophils [37].

In the current study, we included samples from the primary tumor and normal epithelium for some patients. We observed that most samples had high collagen expression independently from the sample's origin, i.e., a normal epithelium or a tumor. In contrast, samples from periampullary adenocarcinoma [11] or colorectal cancer [13], stained in the past by our team with the same method, had noticeable expression of collagen only in a portion of the tumor samples. Thus, the normal levels of collagen expression in esophagus and gastric epithelium appear to be high under normal physiological conditions.

COMP can be measured in patients serum samples by an IVD approved ELISA and is used to assess cartilage turnover in patients with osteoarthritis [38]. Serum COMP levels were prognostic for survival in patients with breast cancer [6], colon cancer [8] and hepatocellular carcinoma [9]. Additionally, a case report described a patient with osteoarthritis who had serum COMP levels twice higher than average. This patients after a month of osteoarthritis therapy was diagnosed with rectal adenocarcinoma following three days of experiencing haematochezia [39]. Cohorts with larger number of patients and the collection of serum samples are needed to clarify the role of COMP in patients with esophageal or gastric adenocarcinoma. Additionally, measuring COMP levels in patient serum and associating them with patient survival could provide a new tool for patients stratification and therapy.

In conclusion, this study provides the initial observation of COMP expression in tumors samples derived from patients with gastric and esophageal adenocarcinoma using immunohistochemistry staining. COMP-expressing tumors had fewer infiltrating immune cells, mainly T-cells, and denser collagen fibers, a hallmark of tumor fibrosis. These observations may indicate that COMP-expressing tumors could be resistant to immune checkpoint inhibitors.

### Supplementary Information

Below is the link to the electronic supplementary material.

Supplementary file 1 (PDF 543 kb)
