# Supplementary material for: The prognostic and potentially immunomodulatory role of cartilage oligomeric matrix protein in patients with gastric and esophageal adenocarcinoma
Source: Cancer Immunol Immunother. 2024 Apr 2;73(5):93. doi: 10.1007/s00262-024-03656-y (PMC10987352; doi:10.1007/s00262-024-03656-y)
Supplement: Supplementary file 1 — (PDF 543 kb) [file 262_2024_3656_MOESM1_ESM.pdf]

Table S1. Expression of COMP in patients with esophageal adenocarcinoma and its association with clinicopathological characteristics.

	Cancer cells					Stroma cells				
**Factor**	**COMP negative**		**COMP positive**		***p*-value**	**COMP negative**		**COMP positive**		***p*-value**
All (N=91)	N	(%)	N	(%)		N	(%)	N	(%)	
Age at diagnosis					0.612					0.822
<50	3	3.3%	0	0.0%		2	2.2%	1	1.1%	
50-70	38	41.8%	12	13.2%		30	33.3%	19	21.1%	
>70	30	33.0%	8	8.8%		21	23.3%	17	18.9%	
Sex					0.408					0.834
Female	9	9.9%	4	4.4%		8	8.9%	5	5.6%	
Male	62	68.1%	16	17.6%		45	50.0%	32	35.6%	
Adjuvant therapy					0.661					0.922
No adjuvant	66	72.5%	18	19.8%		49	54.4%	34	37.8%	
Adjuvant	5	5.5%	2	2.2%		4	4.4%	3	3.3%	
T-stage					0.764					0.297
pT1-T2	16	18.0%	4	4.5%		13	14.8%	6	6.8%	
pT3-T4	53	59.6%	16	18.0%		38	43.2%	31	35.2%	
N-stage					0.464					0.199
pN0	16	17.6%	3	3.3%		13	14.4%	5	5.6%	
pN1-3	55	60.4%	17	18.7%		40	44.4%	32	35.6%	
M-stage					0.308					0.501
M0 or Mx	63	69.2%	16	17.6%		47	52.2%	31	34.4%	
M1	8	8.8%	4	4.4%		6	6.7%	6	6.7%	
Grade					0.655					0.930
Low	43	47.3%	11	12.1%		32	35.6%	22	24.4%	
High	28	30.8%	9	9.9%		21	23.3%	15	16.7%	
R-status					0.742					0.708
R0	46	50.5%	13	14.3%		36	40.0%	22	24.4%	
R1	23	25.3%	7	7.7%		16	17.8%	14	15.6%	
R2	2	2.2%	0	0.0%		1	1.1%	1	1.1%	
Laurén					0.458					0.995
Intestinal	58	63.7%	18	19.8%		44	48.9%	31	34.4%	
Mixed	5	5.5%	0	0.0%		3	3.3%	2	2.2%	
Diffuse	8	8.8%	2	2.2%		6	6.7%	4	4.4%	
Vascular invasion					0.343					0.202
V0	9	24.3%	0	0.0%		8	21.6%	1	2.7%	
V1	22	59.5%	5	13.5%		16	43.2%	11	29.7%	
Uncertain	1	2.7%	0	0.0%		1	2.7%	0	0.0%	
MMR					0.735					0,665
pMMR	65	72.2%	19	21.1%		49	55.1%	34	38.2%	
dMMR	5	5.6%	1	1.1%		3	3.4%	3	3.4%	

Abbreviations: COMP, cartilage oligomeric matrix protein; MMR, DNA mismatch repair; pMMR, proficient MMR; dMMR, deficient MMR. The bold indicates *p*-values <0.05, Calculated with χ^2 two-tailed exact *p*-value.

Table S2. Expression of COMP in patients with gastric adenocarcinoma and its association with clinicopathological characteristics.

Factor	Cancer cells					Stroma cells				
	**COMP negative**		**COMP positive**		***p*-value**	**COMP negative**		**COMP positive**		***p*-value**
All (N=68)	N	(%)	N	(%)		N	(%)	N	(%)	
Age at diagnosis					0.104					0.368
<50	3	4.4%	0	0.0%		3	4.5%	0	0.0%	
50-70	19	27.9%	5	7.4%		19	28.4%	5	7.5%	
>70	39	57.4%	2	2.9%		36	53.7%	4	6.0%	
Sex					0.594					0.946
Female	20	29.4%	3	4.4%		20	29.9%	3	4.5%	
Male	41	60.3%	4	5.9%		38	56.7%	6	9.0%	
Adjuvant therapy					0.548					0.301
No adjuvant	58	85.3%	7	10.3%		56	83.6%	8	11.9%	
Adjuvant	3	4.4%	0	0.0%		2	3.0%	1	1.5%	
T-stage					**0.046**					0.466
pT1-T2	23	33.8%	0	0.0%		20	29.9%	2	3.0%	
pT3-T4	38	55.9%	7	10.3%		38	56.7%	7	10.4%	
N-stage					0.340					0.120
pN0	29	42.6%	2	2.9%		29	43.3%	2	3.0%	
pN1-3	32	47.1%	5	7.4%		29	43.3%	7	10.4%	
M-stage					0.827					0.934
M0 or Mx	54	79.4%	6	8.8%		51	76.1%	8	11.9%	
M1	7	10.3%	1	1.5%		7	10.4%	1	1.5%	
Grade					0.969					0.252
Low	44	64.7%	5	7.4%		41	61.2%	8	11.9%	
High	17	25.0%	2	2.9%		17	25.4%	1	1.5%	
R-status					**0.002**					**0.015**
R0	48	70.6%	2	2.9%		46	68.7%	3	4.5%	
R1	10	14.7%	2	2.9%		8	11.9%	4	6.0%	
R2	3	4.4%	3	4.4%		4	6.0%	2	3.0%	
Laurén					0.333					0.351
Intestinal	31	45.6%	4	5.9%		32	47.8%	3	4.5%	
Mixed	2	2.9%	1	1.5%		2	3.0%	1	1.5%	
Diffuse	28	41.2%	2	2.9%		24	35.8%	5	7.5%	
Vascular invasion					0.611					0.603
V0	6	20.0%	0	0.0%		6	20.7%	0	0.0%	
V1	23	76.7%	1	3.3%		22	75.9%	1	3.4%	
Uncertain	0	0.0%	0	0.0%		0	0.0%	0	0.0%	
MMR					0.344					0.944
pMMR	54	79.4%	7	10.3%		52	77.6%	8	11.9%	
dMMR	7	10.3%	0	0.0%		6	9.0%	1	1.5%	

Abbreviations: COMP, cartilage oligomeric matrix protein; MMR, DNA mismatch repair; pMMR, proficient MMR; dMMR, deficient MMR. The bold indicates *p*-values <0.05, [a] Calculated with χ^2 two-tailed exact *p*-value.

Table S3 Comparison of COMP expression in primary tumors and lymph node metastases

**N=67**	**COMP expression in lymph node metastases**		***p*-value**
**COMP expression in tumor cells**	No (score 0)	Yes (score 1-3)	
No (score 0)	48	2	**0.007**
Yes (score 1-3)	13	4	
**COMP expression in stroma**			
No (score 0)	35	2	**<0.001**
Yes (score 1-3)	26	4	

Abbreviations: COMP, cartilage oligomeric matrix protein; The bold indicates *p*-values <0.05, calculated with the McNemar test.

Table S4 Correlation between COMP, PD-L1 and PD-1 expression in the entire cohort

	Cancer cells (n=157)					Stroma (n=155)				
	**COMP negative**		**COMP positive**		***p*-value**	**COMP negative**		**COMP positive**		***p*-value**
	N	(%)	N	(%)		N	(%)	N	(%)	
**PD-L1 Cancer cells**					0.838					0.631
<1%	100	63.7%	20	12.7%		85	54.8%	34	21.9%	
1-49%	25	15.9%	7	4.5%		20	12.9%	11	7.1%	
≥50%	5	3.2%	0	0.0%		4	2.6%	1	0.6%	
**PD-L1 Immune cells**					0.884					0.773
0-10%	75	47.8%	16	10.2%		65	41.9%	26	16.8%	
11-50%	45	28.7%	9	5.7%		36	23.2%	17	11.0%	
>50%	10	6.4%	2	1.3%		8	5.2%	3	1.9%	
**PD-1 Immune cells**					0.782					0.131
0-10%	65	41.4%	12	7.6%		50	32.3%	27	17.4%	
11-50%	59	37.6%	15	9.6%		54	34.8%	18	11.6%	
>50%	6	3.8%	0	0.0%		5	3.2%	1	0.6%	

Abbreviations: COMP, cartilage oligomeric matrix protein; PD-L1, programmed death-ligand 1; PD-1, Programmed cell death protein. Calculated with Mann–Whitney U two-tailed exact *p*-value. The bold indicates *p*-values <0.05

A

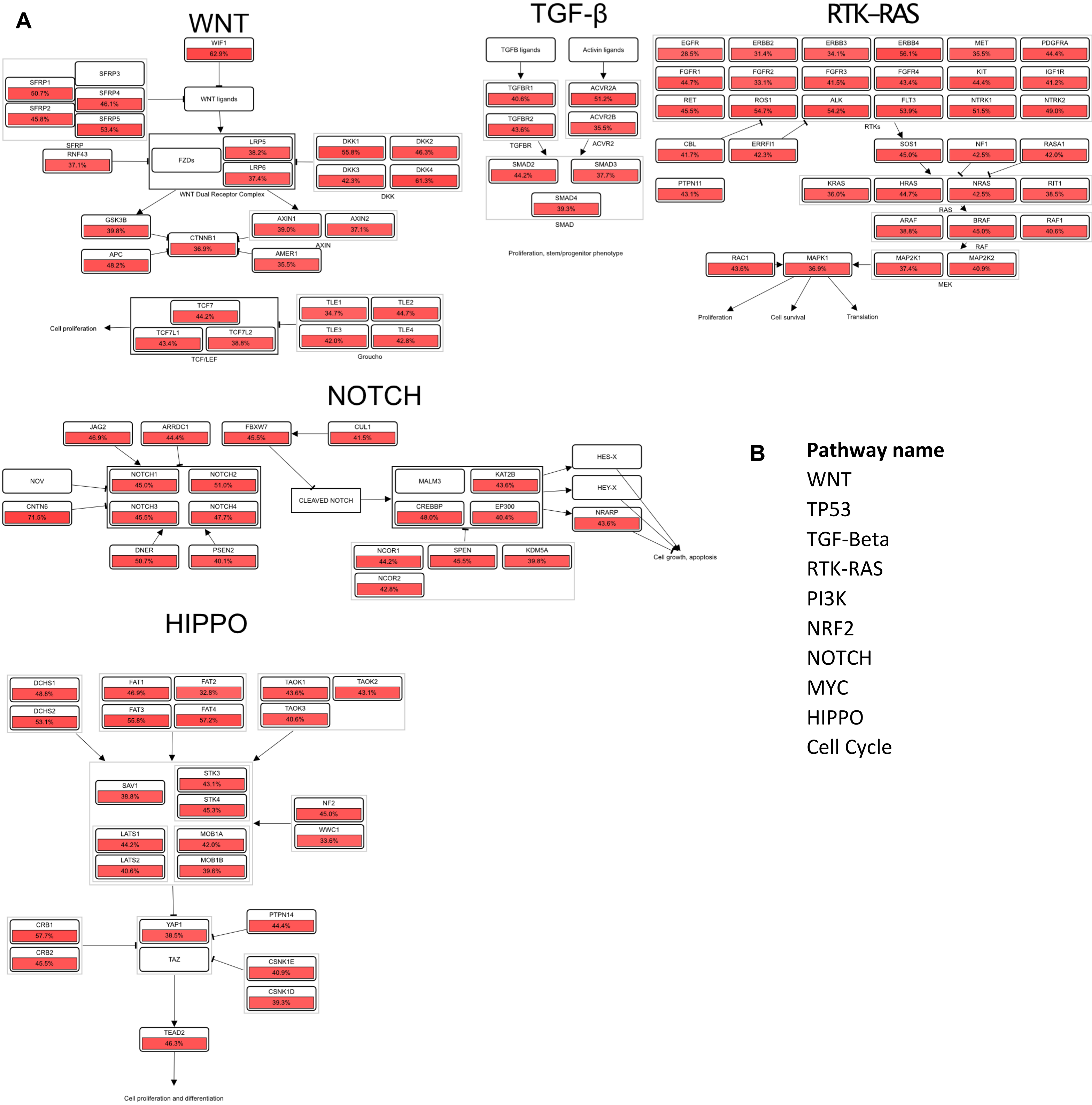

B

Pathway name

WNT
TP53
TGF-Beta
RTK-RAS
PI3K
NRF2
NOTCH
MYC
HIPPO
Cell Cycle

Fig. S1 Pathway analysis was performed with the online tool of the cBioPortal. (**A**) The main affected pathways as presented by the PathwayMapper. The numbers represent the alteration frequencies of the selected gene color-coded using a white to red color scale. (**B**) All the affected gene pathways detected by the analysis are listed.
